# Crop management to enhance plant resilience to abiotic stress using nanotechnology: towards more efficient and sustainable agriculture

**DOI:** 10.3389/fpls.2025.1626624

**Published:** 2025-10-14

**Authors:** Othman Al-Dossary, Lina M. Alnaddaf, Jameel M. Al-Khayri

**Affiliations:** 1 Department of Agricultural Biotechnology, College of Agriculture and Food Sciences, King Faisal University, Al-Ahsa, Saudi Arabia; 2 Department of Field Crops, College of Agriculture, Homs University, Homs, Syria

**Keywords:** crop management, signaling pathways, nanotechnology, abiotic stress, sustainable agriculture

## Abstract

Nanotechnology has become a transformative tool in modern agriculture, playing a pivotal role in enhancing crop resilience to abiotic stresses, including drought, salinity, and extreme temperatures. As global population growth and environmental challenges place increasing pressure on agricultural systems, nanotechnology plays a crucial role in enhancing crop yields and ensuring long-term sustainability. Nanotechnology, through advanced applications, optimizes nutrient delivery, strengthens plant defense mechanisms, and enables precise monitoring of environmental conditions. These innovations enhance soil quality, regulate physiological responses in plants, and mitigate the adverse effects of environmental stressors, thereby promoting sustainable farming practices and improving food production efficiency. Nanoparticles (NPs), synthesized through green methods using plant or microbial extracts, have shown promise in enhancing stress tolerance by facilitating uptake, translocation, and intracellular movement within plants. Major factors influencing NPs efficacy include size, concentration, composition, and duration of exposure. Biosensors and nanobiosensors provide prognostic tools for real-time detection and management of plant stress. Despite their potential benefits, the use of nanotechnology in agriculture raises concerns regarding environmental and health impacts. The accumulation of NPs in soil and aquatic ecosystems may affect microbial diversity, disrupt soil enzymatic activity, and alter plant–microbe interactions, posing risks to non-target organisms and overall ecosystem health. Moreover, variability in plant responses to NPs complicates the development of standardized application protocols. Therefore, its successful adoption relies on sustained interdisciplinary research, ethical oversight, and the development of sound policy frameworks. This integrated approach is crucial for developing resilient, efficient, and sustainable agricultural systems that can meet future challenges.

## 1 Introduction

Global food security faces unprecedented challenges, as the world population is projected to reach 10 billion by 2050, with nearly two billion people currently suffering from nutrient deficiencies and approximately eight hundred million experiencing chronic hunger. Nanotechnology emerges as a revolutionary approach to address these critical challenges, offering innovative solutions that could transform agricultural practices and enhance food production efficiency at the molecular level (Atanda et al., 2025).

Abiotic stresses caused by non-biological environmental factors, including salinity, drought, extreme temperatures, and heavy metal contamination, significantly impact crop yield and global nutritional security, resulting in 20–50% annual global crop yield losses (Cao et al., 2025). Traditional agricultural practices have proven inadequate in effectively mitigating these mounting challenges, necessitating the exploration of cutting-edge technological interventions (Tortella et al., 2023).

The integration of nanotechnology into agriculture represents a paradigm shift, leveraging the unique physicochemical properties of nanomaterials to regulate plant physiological processes, the development of nanofertilizers, nanopesticides, and nanosensors that can optimize nutrient delivery, strengthen plant defense mechanisms, and enable real-time environmental monitoring for sustainable farming practices (Alnaddaf et al., 2025).

Nanoparticles can be strategically introduced into plants through diverse application methods, including seed coating, root uptake, and foliar spraying (Semida et al., 2021). Once internalized, these particles translocate through the vascular system and intercellular spaces to various plant organs, with their uptake and distribution patterns varying based on nanoparticle type and plant species (Zaman et al., 2025).

This targeted delivery system presents a fundamental advantage over conventional agricultural inputs, enabling controlled nutrient release with minimal quantities while maximizing crop yield and reducing environmental impact (Gupta et al., 2023).

Under stress conditions, particularly drought, nanoparticles play crucial roles in maintaining nutritional balance by promoting the absorption, transport, and delivery of essential nutrients such as manganese (Mn), nitrogen (N), zinc (Zn), and potassium (K) within plant tissues (Ahmad et al., 2025).

The transformative potential of nanotechnology extends beyond mere nutrient delivery. Simultaneously, minimizing stress-induced damage by improving soil quality and enhancing stress resistance mechanisms (Shoukat et al., 2025).

### 1.1 Mechanisms of abiotic stress response in plants

Plants perceive abiotic stress via specific sensors located at the cell wall, plasma membrane, cytoplasm, mitochondria, chloroplasts, and other organelles. This stress perception leads to signal transduction pathways involving secondary messengers such as calcium ionsA (Ca^+2^), reactive oxygen species (ROS), and protein kinases, which amplify the stress signal throughout the plant. In addition, stress conditions alter the balance of endogenous plant hormones (Abdelkader et al., 2023). Abscisic acid (ABA) is especially essential for responses to drought and salinity, often mediating stomatal closure to prevent water loss (Francis et al., 2024). Other hormones, such as jasmonic acid and salicylic acid, also play distinct and sometimes combinatorial roles in stress adaptation. Moreover, a network of transcription factors (TFs), including NF-Y, WOX, WRKY, bZIP, and NAC, regulates stress-responsive genes, enabling rapid and targeted genomic adaptation. MicroRNAs (miRNAs) and non-coding RNAs help fine-tune gene expression in stressful environments (Juárez-Maldonado, 2023). Epigenetic changes, including DNA methylation and histone modification, modulate genes that enable stress tolerance. In addition, abiotic stress causes an accumulation of ROS, which can damage plant cells. Plants deploy enzymatic (e.g., superoxide dismutase, catalase, ascorbate peroxidase) and non-enzymatic (e.g., ascorbate, glutathione) antioxidants to neutralize ROS and restore cellular equilibrium (Jósko et al., 2021). As a result of being exposed to various stress factors, plants resort to a set of metabolic and physiological adjustments. These adjustments include modifying nutrient uptake and distribution to maintain a balance of carbon, nitrogen, and phosphorus. Although this balance occurred under prolonged stress, it affects growth and yield. Other adaptive strategies include accumulation of osmolytes (such as proline and sugars), modification of cell membranes, and protein stabilization processes (Khan et al., 2023).

### 1.2 Plant dynamics of abiotic stress

Plants can temporarily repress growth to conserve energy or reallocate resources. These changes may be reversible when stress is alleviated, or they may lead to permanent alterations in development if the stress is prolonged. Plants often face multiple stresses simultaneously, and the interaction between stress pathways can produce unique physiological responses that differ from single-stress reactions (Raliya et al., 2015). Crosstalk between hormonal pathways is central to coordinating these complex responses. Under stress, plants frequently prioritize survival mechanisms over growth, which can result in diminished yield but increased resilience. Plants’ ability to sense, transduce, and respond to abiotic stress is vital for survival and agricultural productivity, especially in the context of climate change and global food security. Understanding and enhancing these mechanisms remain a key focus of plant science and crop improvement research (Thiruvengadam et al., 2024).

### 1.3 The relationship between nanotechnology and plant abiotic stresses

Nanotechnology and plant stress are closely connected through the development and application of nanoscale materials and sensors that help detect, manage, and enhance plant tolerance to abiotic stress via two main points:

Detection and Monitoring: Nano-enabled biosensors and nanobiosensors allow the rapid, sensitive, and real-time detection of plant stress signals, including disease biomarkers, toxins, heavy metals, or stress-related signaling molecules, well before visible symptoms appear. These sensors use advanced nanomaterials (like carbon nanotubes, metal nanoparticles) to greatly improve the precision and speed of stress detection, thus enabling early intervention and smarter crop management (Goyal and Mehrotra, 2025).

Stress Mitigation and Tolerance: Nanomaterials, including green-synthesized nanoparticles of metals such as silver, copper, and zinc, can be delivered to plants to help mitigate effects. Nanoparticles can scavenge harmful reactive oxygen species (ROS) generated during stress, act as stress signaling inducers (boosting a plant’s defenses), and deliver nutrients or protective compounds more efficiently than conventional forms. This can increase tolerance to environmental stresses and reduce crop yield losses (Zaman et al., 2025).

By integrating nanotechnology into agriculture, it is possible to both monitor plant health dynamically and intervene more effectively to raise plant resilience against stress factors. Nanotechnology thus acts as both a diagnostic tool and a direct aid in plant stress management, supporting sustainable and productive agriculture. However, issues with nanomaterial stability, cost, and potential environmental impact remain areas of continued research and debate (Suresh Kumar et al., 2025).

Nanotechnology has a close and growing relationship with abiotic stress management in plants, offering novel tools to detect, mitigate, and enhance tolerance to some non-biological stresses such as drought, salinity, extreme temperature, and heavy metal toxicity (Zaman et al., 2025).

This review comprehensively examines the role of nanotechnology in enhancing plant tolerance to abiotic stresses, addresses key research gaps in the field, and evaluates the broader implications of this emerging technology for achieving more efficient, sustainable, and resilient agricultural systems in the face of global food security challenges.

## 2 Nanotechnology fundamentals

### 2.1 Types of nanoparticles

Nanoparticles (NPs) are recognized for improving nutrient availability and enhancing plant resistance to pathogens and environmental stresses. Various classes of nanomaterials show promise in agricultural applications, including inorganic materials (e.g., silica, copper, iron, zinc, and selenium), organic materials (such as biopolymers, lipids, chitosan, peptides, and proteins), and hybrid materials. Laboratory-synthesized nanoagrochemicals, including nanofertilizers and nanopesticides, have demonstrated a performance increase of approximately 20–30% compared to traditional products (Verma et al., 2024).

Metal and metal oxide nanoparticles, such as silver and zinc nanoparticles, are extensively studied for their effects on secondary metabolism and plant growth. Specifically, zinc oxide nanoparticles (ZnO NPs) have been shown to enhance biomass accumulation and photosynthesis in Arabidopsis (**Table 1**) (Lala, 2021). These nanoparticles promote growth and strengthen plant resilience against abiotic stresses such as drought, cadmium exposure, and salinity. Zinc plays a critical role in the function and stability of various enzymes, supporting the development of healthy crops (Verma et al., 2024). Studies have demonstrated that ZnO NPs significantly improve rice germination rates under salt stress and increase drought tolerance in Kotschy’s dragon head through positive effects on physiological and biochemical traits (Shoukat et al., 2025; Shelar et al., 2024). Furthermore, foliar application of ZnO NPs has resulted in higher chickpea yields than traditional bulk ZnSO_4_ applications, illustrating their capacity to boost plant growth, biomass, and zinc accumulation in grains (Burman et al., 2013).

**Table 1:** Mechanisms of plant resistance to abiotic stresses.

Abiotic stress	Activate plant signaling cascades			Reference
Drought	Enhance stress hormone accumulation example abscisic acid (ABA)	Stress response regulation	Improve water use efficiency	(Heikal et al., 2023)
	affects calcium signaling	Alter Ca^+ 2^ influx or efflux and activating stress response genes	Enhance drought tolerance	(Nasrallah et al., 2022)
Salinity	Increase antioxidant enzyme expressions	Reduce ROS levels and oxidative damage	Enhance SOD and CAT activities	(El-Saadony et al., 2022)
	Influence the salt overly sensitive (SOS) pathway	Stabilize SOS gene expression	Up regulating SOS1 expression	(Mahmoud et al., 2022)
	activate the mitogen-activated protein kinase (MAPK) signaling pathway	Enhance stress-responsive gene transcription	Enhance oxidative stress resistance	(Joshi and Joshi, 2024)
Temperature	Stimulate osmolyte biosynthesis	Aid osmotic adjustment and cellular structure protection	Increase protein accumulation	(Al-Khayri et al., 2023)
	Activate heat shock factors signaling	Increase HSPs accumulation	Increase cellular protection against heat damage	(Yang et al., 2020)
Heavy metal	Modulate the Halliwell–Asada pathway for detoxifying H_2_O_2_	Up regulate pathway enzymes	Reduce H_2_O_2_ levels	(Rajput et al., 2023)

Magnesium oxide nanoparticles (MgO NPs) also contribute to enhanced growth and physiological characteristics in tobacco plants. These nanoparticles increase chlorophyll content, enzyme activity, and magnesium uptake without causing phytotoxic effects (Cai et al., 2020). Additional research has shown that MgO NPs influence growth, chlorophyll content, and gene/miRNA expression in ornamental pineapple. While they enhance plant development at certain concentrations, higher doses may inhibit these benefits (Owusu Adjei et al., 2021).

Polymeric nanoparticles represent another critical category and include cellulose and chitosan nanoparticles. These materials facilitate the delivery of nutrients and protective agents into plant tissues, thereby improving growth and stress resistance. Notably, chitosan nanoparticles have been found to stimulate plant defense mechanisms through complex interactions with physiological pathways (Shinde et al., 2024).

Protein and lipid-based nanoparticles also hold multiple important applications in plant systems. These biocompatible carriers enhance the stability and effectiveness of bioactive molecules during their transport to target sites within the plant. Their application can modulate secondary metabolism and promote the biosynthesis of valuable secondary metabolites (Verma et al., 2024).

As elicitors, nanoparticles have gained considerable attention for their ability to induce the production of secondary metabolites under stress conditions, thereby enhancing plant resistance and metabolic activity. Additionally, they promote the generation of reactive oxygen species (ROS) and trigger the activation of secondary metabolic pathways. This function is particularly important for maximizing the production of commercially valuable bioactive compounds across various industries (Lala, 2021).

Therefore, nanoparticles are increasingly being designed to regulate plant immunity, particularly by interacting with plant-specific pattern recognition receptors (PRRs) and their associated signaling pathways. However, the rigid structure of plant cell walls poses unique challenges to immune modulation via nanoparticles. To overcome this, nanoparticles must be able to penetrate or traverse the cell wall to reach intracellular targets or interact with receptors on the plasma membrane. The formulation or surface modification of nanoparticles is thus critical for enhancing their delivery and uptake by plant cells (Pradeep et al., 2024).

### 2.2 Biogenic synthesis

Biogenic synthesis creates nanoparticles (NPs) sustainably, using plants or microbes instead of harsh chemicals. Plant extracts are a common source. Examples include neem (*Azadirachta indica*) and moringa (*Moringa oleifera*) (Alnaddaf et al., 2021). These extracts contain natural compounds, such as polyphenols and flavonoids, which act as reducing agents. They convert metal salts into NPs. These compounds also act as capping agents, stabilizing the NPs. This method is simple, cost-effective, works at room temperature, and avoids toxic by-products (Murali et al., 2021). Different plants yield NPs of different sizes and shapes. Microbes also synthesize NPs. Bacteria like *Bacillus subtilis* and *Pseudomonas fluorescens* are used. Synthesis can occur inside or outside the bacterial cell. Enzymes or metabolites reduce metal ions. Bacterial synthesis offers good size control and is scalable and eco-friendly (Murali et al., 2023). Fungi and yeast can be used too (Alloosh et al., 2021). Green NPs possess a natural biomolecule coating derived from the plant or microbe. This coating often enhances NP properties, such as biocompatibility or antimicrobial effects. Green NPs are generally safer and show higher efficacy in agriculture than chemically synthesized ones. These methods are crucial, sustainable alternatives (Tasnim et al., 2024).

## 3 Nanoparticle-plant interactions

### 3.1 Mechanisms of nanoparticle internalization and physiological effects in plants

#### 3.1.1 Pathways and transport dynamics of nanoparticles in plants

**Figure 1:**
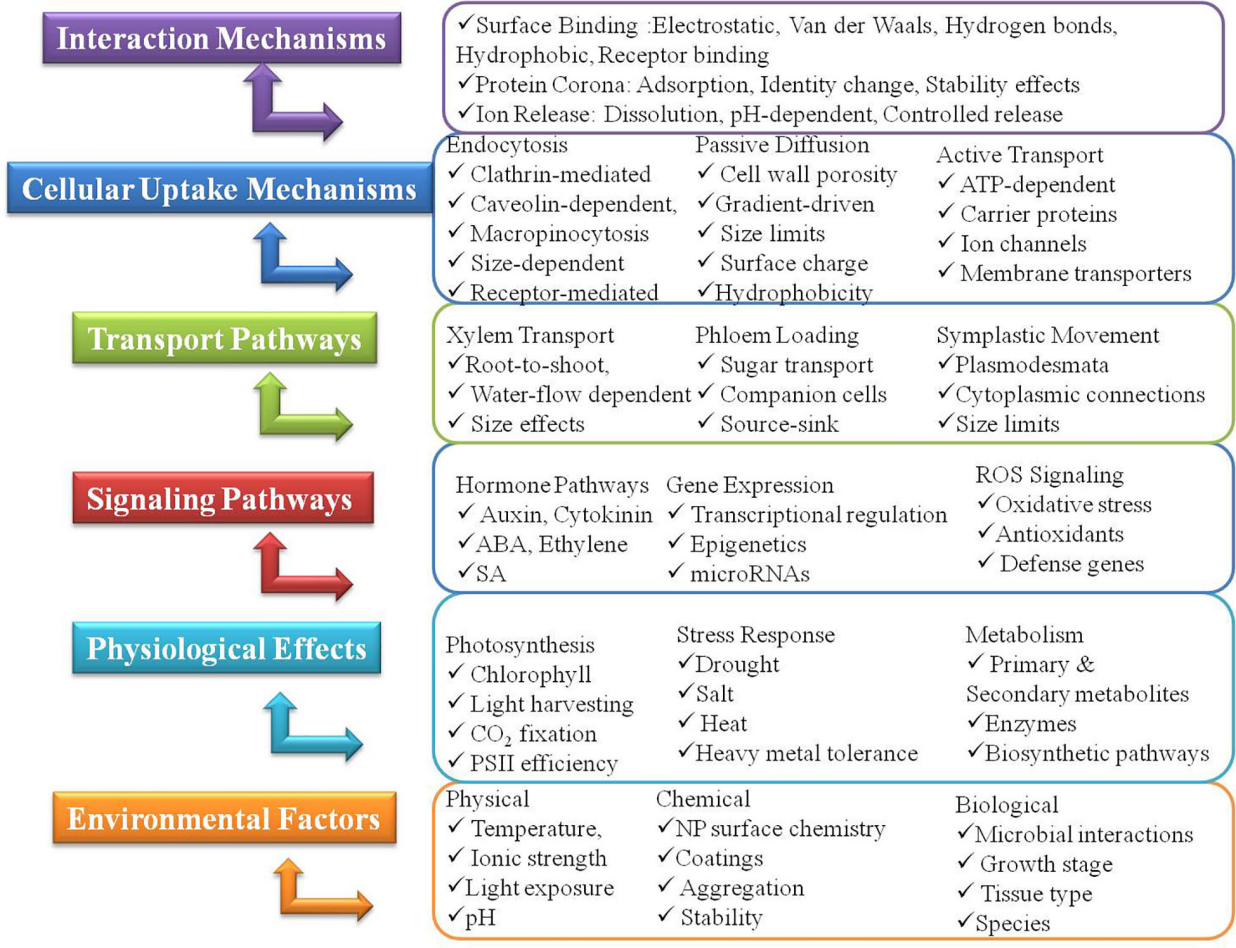
Mechanisms and signaling pathways for nanoparticle application in plants (constructed by L. M. Alnaddaf).

Understanding the intricate processes by which nanoparticles (NPs) enter, move within, and exert their effects on plant systems at both cellular and subcellular levels is paramount for their effective, safe, and sustainable application in agriculture (**Figure 1**) (Alnaddaf et al., 2025).

The primary pathway for nanoparticle entry into plants is through the root system. Nanoparticles can traverse the epidermal cell wall of roots, typically through pre-existing small pores (ranging from 3 to 5 nm). In instances where nanoparticles are larger than these natural pores, they may induce the formation of new entry points, facilitating their absorption (Singh et al., 2024).

Nanoparticles can also enter plants through their leaves, primarily via stomata (microscopic pores on the leaf surface) or through microscopic cracks and imperfections in the leaf cuticle (the waxy protective layer). Various assisted delivery methods, beyond natural entry points, can enhance nanoparticle uptake. These include seed priming (where seeds are treated with nanoparticles before planting), hydroponic systems (where nanoparticles are introduced directly into the nutrient solution), and direct injection methods (Djanaguiraman et al., 2025).

Once inside the plant, nanoparticles can move through the apoplastic pathway. This involves movement through the non-living components, specifically the cell walls and intercellular spaces. This is generally a passive process driven by transpiration pull (Kumar and Biswas, 2023).

Additionally, nanoparticles can move via the symplastic pathway. This involves movement through the living components, specifically the cytoplasm of adjacent cells connected by plasmodesmata (cytoplasmic bridges). This movement often requires the assistance of membrane carrier proteins (Djanaguiraman et al., 2024).

After entering the root or leaf, nanoparticles are efficiently transported throughout the plant via its vascular system, comprising the xylem and phloem. The xylem primarily facilitates the upward transport of nanoparticles from the roots to the aerial parts alongside water and nutrients. The phloem, responsible for sugar transport, can also redistribute nanoparticles throughout the plant, including remobilization back to the roots. The efficiency and pattern of nanoparticle uptake and translocation are significantly influenced by several factors. These include the nanoparticle’s size, shape, surface charge, and chemical composition. Additionally, plant species-specific characteristics and prevailing environmental conditions play crucial roles in determining their movement within the plant (Alnaddaf et al., 2025).

#### 3.1.2 Cellular interactions and physiological impacts of nanoparticles

Nanoparticles initially interact with plant cell walls and plasma membranes, which serve as the primary physical barriers. High concentrations of certain nanoparticles can compromise membrane integrity, potentially leading to the leakage of cellular contents and disruption of normal cellular functions (Gao et al., 2023). Following entry and translocation, nanoparticles can accumulate within various cellular and subcellular organelles. Common sites of accumulation include chloroplasts (affecting photosynthesis), mitochondria (impacting respiration), and vacuoles (involved in detoxification and storage) (Sonkar et al., 2023).

The presence of nanoparticles can induce a wide array of physiological and biochemical changes in plants. These include alterations in photosynthetic rates, nutrient uptake efficiency, enzyme activity, and overall gene expression patterns (Alnaddaf et al., 2025).

While some nanoparticles mitigate oxidative stress by enhancing antioxidant defense systems, others, particularly at elevated concentrations, can paradoxically induce oxidative stress by promoting the generation of reactive oxygen species (ROS). Nanoparticles have been observed to influence the synthesis, transport, and signaling pathways of key plant hormones, such as auxins, gibberellins, cytokinins, and abscisic acid. This modulation can significantly impact plant growth, development, and adaptive stress responses (Páramo et al., 2023; Selvakesavan et al., 2023).

Emerging research suggests that nanoparticles may interact with the plant’s genetic material, potentially leading to changes in gene expression or, in some cases, DNA damage. Further research is needed to fully understand these complex genetic interactions and their long-term implications (Alnaddaf et al., 2023).

Crucially, the effects of nanoparticles are highly dose-dependent. Low and optimized concentrations often confer beneficial effects, such as enhanced growth, improved nutrient utilization, and increased stress tolerance. Conversely, higher concentrations can lead to phytotoxicity, growth inhibition, and other adverse impacts on plant health (Murali et al., 2022).

### 3.2 Determinants of nanoparticle efficacy and various effects on plants

The impact of nanoparticles on plants is a complex phenomenon, highly contingent upon a multitude of factors related to the nanoparticles themselves, the specific plant species, and the prevailing environmental conditions. A thorough understanding of these influencing factors is paramount for optimizing the beneficial applications of nanotechnology in agriculture while simultaneously mitigating potential risks (**Tables 2**, **3**).

**Table 2:** Potential effects of NPs on plant growth, physiology, nutrition and stress mitigation.

Nanoparticle type	Potential effects	Reference
Enhanced growth & nutrient uptake		
ZnO and Fe_2_O_3_	Zinc oxide and iron oxide (Fe_2_O_3_) nanoparticles enhance seed germination rates by promoting enzymatic activity and boosting energy production within seeds.	(Guo et al., 2022)
ZnO NPs	stimulate root elongation and lateral root formation increasing the availability of essential micronutrients	(Nair and Chung, 2017)
ZnO NPs	Enhance zinc ion availability, essential for enzyme function and protein synthesis, improving plant nutrition and growth, especially under nutrient-limited conditions.	(Rashid et al., 2023)
Graphene Oxide	Act as carriers for nutrients like magnesium, further boosting photosynthetic activity.	(Santana et al., 2022)
Carbon-based nanomaterials CNTs	Form nanochannels in root cell membranes, facilitating water/nutrient transport and supporting robust root/shoot development in seedlings.	(Joshi et al., 2018)
Cu and Zn NPs	Boost enzymatic activities related to carbohydrate metabolism, ensuring sufficient energy for growth.	(Malik et al., 2021)
Fe_2_O_3_ NPs	Improve root/shoot biomass (e.g., in wheat, rice) by providing a readily absorbable iron source, facilitating efficient iron uptake and utilization, and addressing iron deficiency.	(Hussain et al., 2019)
TiO_2_ NPs	Enhance seed germination by improving water uptake and accelerating the breakdown of stored carbohydrates. This results in faster, uniform seedling emergence.	(Shah et al., 2021)
ZnO NPs	Improve zinc (Zn) ion availability, which is essential for enzyme function and protein synthesis	(Jan et al., 2021)
Ag NPs	Promote shoot growth by enhancing cell division and elongation in the apical meristem	(Wang et al., 2019)
Enhanced photosynthesis		
TiO_2_ NPs	Increase light absorption and chlorophyll content, leading to higher photosynthetic rates. They interact with chloroplasts to improve the efficiency of light-dependent reactions and carbon fixation.	(Dias et al., 2018)
Stress mitigation		
Reducing Oxidative Stress (ROS Scavenging)		
Carbon-based Nanomaterials (CNTs)	Reduce oxidative stress by scavenging ROS, stabilizing metabolic processes under stress. stabilizing metabolic processes under challenging environmental conditions	(Patel et al., 2024)
ZnO and TiO_2_	Reduce oxidative damage by scavenging ROS, preventing cellular degradation during water scarcity.	(Khan et al., 2015)
Au and Ag	Reduce heat-induced ROS accumulation, shielding plants from oxidative damage.	(Wu et al., 2017)
ZnO and cerium oxide (CeO_2_)	Reduce ROS accumulation caused by heavy metal toxicity, minimizing oxidative damage	(Xia et al., 2008)
Salinity stress		
Ag NPs	Mitigate ion toxicity by reducing sodium uptake while increasing potassium assimilation, preserving ionic homeostasis	(Khan et al., 2020)
Silicon (Si) NPs	Strengthen cell walls and improve membrane stability, mitigating salt-induced dehydration	(Wei et al., 2015)
carbon-based nanomaterials	Promote osmolyte (e.g., proline) production, maintaining cellular turgor and enzymatic activity, enhancing resilience under salinity.	(Safikhan et al., 2018)
Heavy metal stress		
Fe_2_O_3_ NPs	Exhibit high affinity for heavy metal ions, immobilizing them in soil and preventing plant uptake	(Soliemanzadeh and Fekri, 2021)
Heat stress		
Si NPs	Stimulate heat shock protein (HSP) production, protecting cellular proteins and membranes from denaturation	(Haq et al., 2019)

#### 3.2.1 Nanoparticle properties: uptake and bioactivity

The concentration at which nanoparticles are applied is a critical determinant of their effects. Low, optimized concentrations often elicit beneficial responses, such as enhanced plant growth, improved nutrient uptake, and increased tolerance to various stresses. Conversely, higher concentrations can lead to phytotoxicity, induce oxidative stress, and impair essential physiological processes (Siddiqi et al., 2021).

Moreover, the physical size of nanoparticles profoundly influences their uptake, subsequent translocation within the plant, and their interactions with plant cells. Generally, smaller nanoparticles (e.g., those in the 3-5 nm range) are more readily absorbed by roots and exhibit greater mobility throughout the plant vascular system (Khan et al., 2022). The size exclusion limits of plant tissues, such as the pores in the cuticle and stomata, play a crucial role in regulating nanoparticle entry. Furthermore, the morphology or shape of nanoparticles can also significantly affect their cellular uptake and interactions with plant cells. Different shapes may influence their biological activity and potential toxicity (Tripathi et al., 2021).

The surface charge of nanoparticles is a key factor influencing their initial interaction with plant surfaces (e.g., the root epidermis) and their subsequent movement within plant tissues. Surface modifications, which can alter the charge, are often employed to enhance uptake efficiency or achieve targeted delivery (Nair et al., 2020).

The intrinsic chemical composition of nanoparticles dictates their inherent properties and how they interact with biological systems. Nanoparticles composed of different materials (e.g., silver, gold, zinc oxide, and titanium dioxide) can exert distinct effects on plant physiology and biochemistry (Rajput et al., 2021).

In addition, the presence of a coating or specific surface modifications on nanoparticles can significantly alter their stability, dispersibility in various media, uptake efficiency, and overall biological activity. These modifications are strategically used to enhance beneficial effects or reduce potential toxicity (Alnaddaf et al., 2025).

#### 3.2.2 Exposure parameters: duration, application method, and environmental context

The duration of plant exposure to nanoparticles directly influences the extent of nanoparticle uptake, their accumulation within plant tissues, and the resulting physiological responses. Prolonged exposure may lead to different outcomes compared to acute, short-term exposure (Sharma et al., 2020). The application method (e.g., foliar spray, direct soil application, seed treatment, or integration into hydroponic systems) dictates the primary entry pathways and subsequent distribution patterns within the plant (Ahmad et al., 2023). External environmental conditions, including soil pH, ambient temperature, light intensity, and the presence of other ions or compounds in the soil or growth medium, can significantly influence nanoparticle stability, bioavailability to plants, and interactive effects with plant systems (Yasmeen, 2023).

#### 3.2.3 Plant species-specific and developmental stage-dependent responses

Different plant species exhibit varying degrees of sensitivity and diverse physiological responses to nanoparticles. These variations are due to differences in inherent physiological characteristics, root architecture, leaf morphology, and unique genetic makeup (Zhang et al., 2022). Plant root exudates (organic compounds released by roots into the rhizosphere) can interact with nanoparticles in the soil. This interaction can affect nanoparticle aggregation, dissolution, and ultimately, their uptake (Chen et al., 2021). The specific developmental stage of the plant at the time of nanoparticle exposure influences its susceptibility to nanoparticle effects and its capacity to respond adaptively (Zhou et al., 2023).

Understanding the interplay of these complex factors is essential for the rational design and development of effective and safe nano-agricultural products, as well as for accurately predicting their environmental fate and overall impact on agricultural ecosystems.

## 4 Mechanistic roles of NPs in abiotic stress mitigation

Nanoparticles play pivotal roles in alleviating plant stress through multiple interconnected mechanisms that enhance the plant’s intrinsic defense systems and optimize key physiological processes, thereby sustaining plant health and productivity under challenging environmental conditions (**Table 1**, **Figure 1**) (Zaman et al., 2025). Specifically, we examine how nano-formulated nutrients, in conjunction with phytohormones, influence plant growth and stress resilience. This influence occurs through the promotion of antioxidant enzyme synthesis. These nano-enabled strategies activate critical defense mechanisms, including reactive oxygen species (ROS) scavenging systems, thereby improving plant tolerance to adverse environmental conditions.

Our analysis demonstrates how nano-mineral nutrient management simultaneously modulates key antioxidant enzymes (superoxide dismutase, catalase, and peroxidase) and regulates phytohormonal activity (auxins, cytokinins, and gibberellins). This dual modulation is necessary for three key processes: reducing ionic toxicity, strengthening oxidative stress responses, and maintaining cellular water balance in stressed plants (Meng et al., 2025).

### 4.1 Drought stress mitigation and associated mechanisms

Drought is one of the most critical abiotic challenges facing global agriculture, resulting from insufficient irrigation and reduces rainfall that leads to prolonged dry periods, severely limiting crop growth. The complex nature of drought stress makes it difficult to monitor and manage effectively through conventional approaches (Pérez-Labrada et al., 2020).

#### 4.1.1 Mechanistic responses to drought

Recent studies demonstrate that cerium oxide nanoparticles enhance drought tolerance in sorghum by regulating abscisic acid (ABA)-related genes and promoting the activity of antioxidant enzymes (El-Saadony et al., 2022). ABA plays a vital role during drought stress by promoting stomatal closure to minimize water loss and interacting with JA/SA signaling pathways. The P5CS gene enhances proline biosynthesis for osmotic adjustment under drought, while AREB/ABF transcription factors activate ABA-responsive genes during water stress (Yoshida et al., 2015). Downregulation of TAS14 and ZFHD genes increases drought tolerance by regulating ABA biosynthesis and osmotic pressure (Pérez-Labrada et al., 2020).

Silver nanoparticles (AgNPs) alleviate drought stress in plant species such as lentils by maintaining water balance and enhancing growth parameters through improved osmotic adjustment and enhanced antioxidant capacity (Ahmad et al., 2024). Iron nanoparticles (FeNPs), particularly Fe_2_O_3_, play vital roles in several plant metabolic processes, including photosynthesis, respiration, DNA synthesis, and pigment production. Fe_2_O_3_ nanoparticles can mitigate oxidative stress in drought-exposed plants by reducing reactive oxygen species (ROS) levels and enhancing chlorophyll content, thereby improving photosynthetic efficiency under stress conditions (Shang et al., 2019).

### 4.2 Salinity stress mitigation and ionic homeostasis

Salinity affects approximately 20% of the world’s arable land, arising from high sodium chloride concentrations in coastal and arid regions. This condition particularly threatens glycophytes, which are highly sensitive to salt stress, limiting both food production and crop quality (Cao et al., 2025).

#### 4.2.1 Mechanistic responses to salinity

Iron oxide nanoparticles enhance plant growth in saline environments by helping maintain ionic balance through activation of salt overly sensitive (SOS) kinases and calcium signaling pathways, which regulate gene networks for stress mitigation and ion homeostasis (Meel and Saharan, 2024). These pathways involve mitogen-activated protein kinases (MAPKs) that initiate defense responses and facilitate cellular repair processes.

Zerovalent nano-iron (nZVI) particles may release hydroxyl radicals (OH•) capable of degrading pectins. Zinc oxide nanoparticles mitigate the excessive salinity effect on basil plants by regulating proline accumulation and enhancing antioxidant activity (Cao et al., 2025). Silver nanoparticles (AgNPs) alleviate salt stress by restoring ionic balance and improving nutrient availability, with studies showing nearly doubled growth parameters in quinoa Q6 line exposed to salinity stress (Gupta et al., 2023).

### 4.3 Temperature stress and physiological protection

Temperature represents another major risk factor affecting crop yields, with high temperatures negatively affecting respiration, transpiration, and photosynthesis. Temperature stress leads to reduced photosynthesis, shortened growth periods, and ultimately lower crop yields. Challenges intensify under climate change conditions (Sidhu et al., 2024).

#### 4.3.1 Mechanistic responses to temperature stress

Nanoparticles provide physical protection by enhancing the structural integrity of plant cell walls, making plants more resilient to thermal and environmental stresses. They also modulate levels of key phytohormones such as ethylene (ET), which regulates genes associated with cell wall reinforcement and defense activation under temperature stress conditions (Sidhu et al., 2024).

### 4.4 Heavy metal stress and detoxification mechanisms

Heavy metals, soil erosion, floods, and nutrient deficiencies considerably affect crop yield and quality. Silicon nanoparticles (SiNPs) reduce cadmium (Cd) stress by increasing biomass and lowering oxidative stress through enhanced antioxidant enzyme activities, including superoxide dismutase (SOD), glutathione reductase (GR), catalase (CAT), and phenylalanine ammonia-lyase (PAL) (Ahmed et al., 2023).

#### 4.4.1 Mechanistic responses to heavy metal toxicity

SiNPs significantly improve both yield and quality of rice grains under lead (Pb) and Cd stress by inhibiting metal uptake and facilitating sequestration into less harmful cellular compartments (Khanna et al., 2021). Iron nanoparticles (FeNPs) stimulate growth in stressed plants by promoting chlorophyll content and photosynthesis while reducing the toxicity and metal bioavailability in contaminated soils (Naidu et al., 2023).

### 4.5 Molecular signaling pathways and stress response integration

#### 4.5.1 ROS regulation and antioxidant defense

NPs can induce controlled oxidative stress by increasing ROS levels, which may initially disrupt redox homeostasis (**Table 1**). However, they simultaneously enhance antioxidant defense systems by stimulating both enzymatic antioxidants (SOD, CAT, APX, GR) and non-enzymatic antioxidants (ascorbic acid, glutathione, phenols, flavonoids). Some nanoparticles, like C_60_ fullerenes, directly scavenge excess ROS, reducing oxidative damage during abiotic stress (Pandey et al., 2018).

#### 4.5.2 Hormonal regulation and gene expression

NPs modulate levels of key phytohormones, including ABA, jasmonic acid (JA), and salicylic acid (SA), which are central to abiotic stress signaling. JA regulates genes for defense compounds and secondary metabolite production, while SA coordinates systemic acquired resistance responses (Ahmad et al., 2024). Bionanoparticles can deliver nucleic acids into plant cells, leading to upregulation or downregulation of specific genes related to stress adaptation through interactions with cellular components that affect gene accessibility (Bahwirth et al., 2023).

#### 4.5.3 Secondary metabolite enhancement

NPs act as elicitors, triggering ROS production that activates secondary metabolic pathways, enhancing synthesis of bioactive compounds, such as flavonoids, phenolics, and terpenoids. These compounds serve dual roles in plant defense and human health benefits. Exposure to copper oxide nanoparticles (CuO) increased polyphenol content and antioxidant activity in Withania somnifera (Raza et al., 2024).

#### 4.5.4 Nutrient optimization and physical protection

As nanofertilizers, NPs enhance nutrient uptake and utilization efficiency, ensuring adequate nutrition during stress conditions while supporting overall metabolism and growth. They also aid in osmotic adjustment by promoting osmotic accumulation, helping plants maintain turgor and essential physiological functions under water-deficient conditions (Saleh et al., 2021).

The multifunctional nature of nanoparticles, from molecular signaling to whole-plant physiological responses, underscores their significant potential in enhancing plant resilience to abiotic stresses. Their ability to simultaneously address multiple stress factors while optimizing plant defense mechanisms positions nanotechnology as a promising tool for advancing sustainable and stress-resilient agriculture under changing climate conditions (Alnaddaf et al., 2025).

## 5 Agricultural applications

### 5.1 The role of nanotechnology in promoting crop resilience against abiotic stress

Nanoparticles utilize their diverse and versatile properties to enhance plant tolerance to abiotic stresses, affecting all parts of the plant through multiple complementary mechanisms (**Tables 2**, **3**).

**Table 3:** Effect of nanoparticles on different plants.

Nanoparticle type	Concentration	Mode of application	Plant	Remarks	Reference
nano-Si-CDs	10 mg/L	foliar	Maize	Significantly increased fresh weight (327.1% in roots, 247.2% in shoots) and dry weight (212.0% in roots, 118.5% in shoots)	(Yao et al., 2023)
carbon dots (CDs)	10, 20, 30, and 40 mg/L	fertilizer	Lettuce	Enhanced photosynthetic activity by improving key mechanisms, including chloroplast activity, chlorophyll content, Rubisco enzyme activity, and photosystem II (PSII) performance.	(Hu et al., 2022)
Si NPs	–	fertilizer	Radish	Boosted fresh weight (36.0%), chlorophyll content (14.2%), and carotenoid levels (18.7%); increased reducing sugars (23.7%), total sugars (24.8%), and proteins (232.7%), enriching nutritional profile	(Xu et al., 2023)
Ag NPs	10 or 20 mg/L	soaking	Rice	Enhanced germination, seedling growth, water uptake, and upregulated aquaporin genes (PIP1;1, PIP2;1); elevated amylase, dehydrogenase, and catalase activity.	(Mahakham et al., 2017)
ZnO NPs	2–9 g/L	foliar	Saffron	Increased flower yield, chlorophyll content, water retention, protein levels, and antioxidant enzyme activity (POX, CAT)	(Rostami et al., 2019)
TiO_2_ NPs	–	foliar	Vetiveria zizanioides	Enhanced biomass, essential oil/khusimol production, chlorophyll, PSII efficiency, nitrate reductase, and carbonic anhydrase activity	(Shabbir et al., 2019)
CeO_2_:SA NPs	–	foliar	*Portulaca oleracea* L.	Reduced salt stress impact on purslane through improved physiological functions and growth-related characteristics.	(Hassanpouraghdam et al., 2021)
Fe NPs 54nm	500mg/kg	fertilizer	Arabidopsisthalian	Increased plant biomass, carbohydrates, and phosphorus via enhanced photosynthesis (increased stomatal opening) and improved P availability (reduced rhizosphere pH).	(Yoon et al., 2019)
Fe NPs	20 mg/L	soaking for three days	Rice	Improved seedling growth was achieved by increasing tissue water content, boosting the activity of hydrolytic and antioxidant enzymes, strengthening cell membrane integrity and viability, and elevating chlorophyll and iron levels.	(Guha et al., 2018)
ZnO NPs	10 mg/L	foliar	Mung bean	Promoted overall plant growth (resulting in longer stems and larger root volume), improved nutrient uptake (specifically phosphorus accumulation by stimulating phosphatase and phytase activity), increased photosynthetic capacity (via higher chlorophyll and protein levels), and fostered beneficial rhizosphere microbial populations.	(Raliya et al., 2016)
Fe NPs	25 mg/kg	fertilizer	Wheat	Enhanced growth more substantially than in normal soil, increasing root/shoot/grain dry weights and outperforming FeSO_4_/Fe-EDTA	(Zia-ur-Rehman et al., 2023)
Fe-0 NPs (35–45 nm)	8–14 m²/g	fertilizer	Sunflower	Alleviated stress via soil Cr immobilization & upregulated antioxidant enzymes (SOD, POD, CAT, APX)	(Mohammadi et al., 2020)
ZnO NPs	25 ppm	seed priming	Rice	Reduced oxidative damage, enhanced antioxidant activity & proline	(Mazhar et al., 2022a)
SeNPs	75 ppm	seed priming	Tomato	Lowered oxidative stress, boosted antioxidant defense & ascorbate-glutathione cycle, increased bioactive compounds	Ishtiaq et al., 2022)
CaONPs	75 ppm	seed priming	canola	Improved germination (30%), seedling fresh weight (34%), leaf number (16%), chlorophyll (28.9%), pod/seed production (73%), 100-seed weight (35.13%), yield (35.18%) via improved antioxidants & reduced stress markers.	(Mazhar et al., 2022b)
CuNPs	20mg/L	Foliar	Trigonella foenum-graecum L.	Improved growth/biomass, promoted pigments, osmolytes, anthocyanin, shikimic acid, phenols, upregulated antioxidant enzymes	(Fouda et al., 2024)
ZnO NPs	20, 40, and 60 mg/L for 12 h	soaking	(Lupinustermis) seeds	Mitigated negative impacts of 150 mM NaCl stress on seedlings.	(Hossain et al., 2021)
Fe_2_O_3_ NPs (40 nm)	5, 10, 20, 30, and 40 µM	fertilizer	lemon balm (Melissa officinalis	Increased essential oil, restored chlorophyll, decreased proline, MDA, H_2_O_2_	(Mohasseli et al., 2020)
γ-Fe_2_O_3_ NP	0.5, 0.8, 1, or 2 mg/mL	fertilizer	Brassica napus	Increased chlorophyll, growth, reduced H_2_O_2_	(Palmqvist et al., 2017)
nanochitosan	(0.05% and 0.1%)	seed priming	broad bean seeds	Inhibited germination/seedling growth due to cytotoxicity; NPs penetrated seed coat, accumulated in embryos, disrupting cell division/development.	Abdel-Aziz, 2019
nano-biochar	–	fertilizer	various crops	Decreased cumulative runoff (10.88–31.75%) and sediment; increased rainfall interception (18.08–31.14%) on slopes	Chen et al., 2020
Schiff base -Nanoclay Polymer Composites (NCPCs)	–	fertilizer		Effectively inhibited nitrification (30–87%); slowed nitrification & reduced nitrate leaching	Saurabh et al., 2019
slow-release nanoclay polymer composites made up of acrylic acid (AA) and acrylamide (Am)	–	fertilizer	Rice, wheat	Improved N-use efficiency: Reduced N_2_O emissions (rice: 16.1%; wheat: 12.4%) vs. urea	Kirti et al., 2021
macro-nutrient (K_2_SO_4_) and micro-nutrient (ZnO and SiO_2_)	salt concentrations (6 and 10 dS/L^)^	hydroponic experiment	*Medicago sativa* L.	Improved plant height, shoot dry weight, flowers, tillers, root length, root fresh/dry weight (nano-K_2_SO_4_); enhanced proline/SOD under salinity (nano-SiO_2_)	El-Shal et al., 2022
AgNPs	20-30 ppm	fertilizer	Soybean	Enhanced *Bradyrhizobium japonicum* symbiotic efficiency (25-30%), improving nodulation & N-fixation.	(Siddiqui et al., 2021)
AgNP-treated Mesorhizobium ciceri inoculants	–	fertilizer	Chickpea	Reduced *Fusarium oxysporum* infections (40%) while boosting growth.	(Abd-Alla et al., 2020).
Fe_2_O_3_ NPs	–	fertilizer	Wheat	Synergy with *Bacillus megaterium*: 35% higher grain Fe, 28% yield increase vs. traditional fertilization.	(Zhao et al., 2021).
ZnO NPs combined with zinc-mobilizing Pseudomonas brassicacearum (20-30 nm)	–	cultivation in zinc-deficient paddies	Rice	Increased Zn uptake efficiency (45-50%), addressing malnutrition	(Zhang et al., 2022).
graphene oxide (GO) sheets functionalized with plant growth-promoting Bacillus amyloliquefaciens	–	hydroponic	Lactuca sativa	Enhanced nutrient uptake efficiency (35-40%), reduced leaf nitrate accumulation	(Torres et al., 2022)
SiO_2_ NP-coated Azospirillum brasilense inoculants	–	In drought-prone	Sorghum	Maintained 75% colonization efficiency at 40% field capacity (vs. 30% uncoated).	(Nguyen et al., 2023)
silica-encapsulated Halomonas venusta biofertilizers	–	Saline coastal areas	Rice	0% higher survival & maintained nitrogenase activity at 8 dS/m salinity.	(Wang L. et al., 2023).
chitosan nano-formulations containing Trichoderma harzianum	–	fertilizer	Grapevine	Provided season-long root pathogen protection & enhanced nutrient uptake	(Martínez et al., 2023).
Fe2O3 NPs	–	fertilizer	Pumpkin	45% accumulated in roots, <1% detected in leaves after root uptake & translocation.	(Zhu et al., 2008)
fullerene (C70)	–	foliar		Transported root→shoot and leaf→root (via phloem)	(Lin and Xing, 2008)
CuO NPs	100 mg/L	fertilizer	Maize	Found in epidermis cell walls, intercellular spaces, cortical cell cytoplasm/nuclei; suggesting apoplastic transport.	(Wang X. et al., 2023)
ZnO NPs	a high concentration	fertilizer	Tomato	Induced tomato oxidative stress, reducing growth/biomass.	(Li et al., 2016)
Ag NPs	–	fertilizer		Penetrated cell walls, damaging morphology	(Sangour et al., 2021)
Ag NPs	–	fertilizer	kiwi	Caused pollen mortality via membrane damage	(Speranza et al., 2013)
Ag NPs	higher concentrations	fertilizer	*A. thaliana*	Reduced shoot/root length by destroying root apical meristem cells	(Wang et al., 2013)
Ag NPs		fertilizer	Mung beans	Generated ROS, causing lipid peroxidation & cellular damage.	(Nair and Chung, 2015)
TiO_2_ NPs	500 mg/L to 4000 mg/L	fertilizer	*Brassica napus L*	Improved morphology/physiology (root length, height, biomass, gas exchange, chlorophyll, NRase) up to 4000 mg/L foliar, but also caused pollen mortality.	(Li et al., 2015)
ionic cerium, bulk CeO_2_, and CeO_2_ NPs	10 mg/L	fertilizer	Radish	The nanoparticles exhibited a range of effects, including negative, positive, and instances where no significant impact was observed.	(Zhang W. et al., 2015)
CeO_2_ NPs (lab-synthesized 7 nm and 25 nm and commercial CeO_2_ NPs)	-	fertilizer	Latuca species	All three tested CeO_2_ nanoparticle types demonstrated toxicity towards the three *Lactuca* species studied. Furthermore, the different CeO_2_ nanoparticles exhibited varying levels of toxicity.	(Zhang P. et al., 2015)
CeO_2_ NPs (~250 nm in size) and bulk CeO_2_ particles (~2000 nm in size)	–	fertilizer	*Brassica napus* L.	Application of bulk CeO_2_ at concentrations of 10 mg/L and 100 mg/L increased plant biomass by 28% and 35%, respectively. In contrast, CeO_2_ nanoparticles applied at these same concentrations did not produce this enhancing effect.	(Ma et al., 2015)
CeO_2_ NPs	–	fertilizer	Rice	CeO_2_ nanoparticles altered the root’s antioxidant defense system. Specifically, at a low concentration (62.5 mg/L), they inhibited root H_2_O_2_ production by 75%. Conversely, at higher concentrations (125 mg/L and 500 mg/L), they significantly elevated root H_2_O_2_ levels. This increase led to lipid peroxidation and electrolyte leakage.	(Rico et al., 2013)
ZnO NPs	50 and 100 ppm	Foliar	eggplant	improve physiological traits, including membrane stability index (MSI) and relative water content (RWC), enhance photosynthesis, and strengthen the anatomical structure of stems and leaves. Critically, they also increase fruit yield by 12.2% and 22.6% (at 50 ppm and 100 ppm, respectively) compared to untreated plants.	(Semida et al., 2021)
ZnO NPs	50 or 100	60% ETc irrigation	eggplant	Treated plants achieve 50.8–66.1% higher WP than fully irrigated plants without nanoparticle treatment.	(Semida et al., 2021)
FeNPs	100 mg/L	Foliar	Tomato	Application significantly enhances growth, increasing shoot length (42%), root length (66%), fruit weight (24%), fruit number (66%), leaf number (173%), and branch number (45%). FeNPs also boost pigment content (carotenoids and lycopene) and reduce cadmium accumulation in plant tissues	(Ahmad et al., 2024)

#### 5.1.1 Enhanced nutrient delivery and smart release systems

Nanotechnology enables the development of NP-fortified fertilizers that deliver nutrients in a targeted manner, minimizing nutrient runoff and reducing groundwater contamination risks (Ahmad et al., 2024). Integration with smart fertilizers allows programmed nutrient delivery based on specific environmental conditions, such as soil moisture or pH fluctuations, supplying crops with necessary nutrients at optimal times (Pradeep et al., 2024).

Nanofertilizers coat or encapsulate nutrient substances, allowing controlled release into the soil while helping recharge depleted soils and maintain soil health (Salem et al., 2021). Nitrogen, potassium, and phosphorus nanoparticles improve macronutrient utilization and availability, resulting in better plant growth and increased stress tolerance (Khanna et al., 2021).

#### 5.1.2 Rhizosphere interactions and soil enhancement

NPs interact multifacetedly with the rhizosphere, contacting microbes, minerals, and organic matter that indirectly affect plant roots. Their biological inertness allows persistence in soil for extended periods, causing significant changes in microflora populations, soil fertility, and plant physiology (Meel and Saharan, 2024).

When incorporated into soil, NPs enhance aeration and water-holding capacity, facilitating better gas exchange and water absorption. They stimulate soil enzyme activity, sustaining soil health and boosting organic processes essential for nutrient cycling (El-Saadony et al., 2022). NPs also promote plant growth-promoting microorganisms (PGPM) such as Rhizobium and Bradyrhizobium, which form symbiotic associations that fix nitrogen and mobilize nutrients (**Table 4**).

**Table 4:** The role of plant growth-promoting microorganisms in drought tolerance.

Plant growth-promoting rhizobacteria (PGPR)	Mechanisms of rhizobacteria-induced drought tolerance in plants		References
*Lactobacillus paracasei*, *Rhodococcus jostii* RHA1, *Microbacterium* sp. 3J1, *Arthrobacter siccitolerans* 4J27, *Rhodococcus* sp. 4J2A2, *Rhodococcus opacus* PD630, and *Pseudomonas putida KT2440*	Overproduce varieties stable proteins and osmolytes	Balance the available water or reduce water loss	(Yerbury et al., 2005; Palud et al., 2020; LeBlanc et al., 2008)
	Increase gene expression that codes for enzymes involved in DNA synthesis	Reduce plant damage	(García-Fontana et al., 2016; Narváez-Reinaldo et al., 2010; Alvarez et al., 2004)
	Activate drought-sensitive genes	During drought period, plants decrease evaporation and enhance water absorption via reducing leaves size and extending roots further into the soil.	(Wang et al., 2018; Vílchez et al., 2016)

#### 5.1.3 Advanced agricultural applications

Nanopesticides: Developed to improve pest control efficiency by delivering pesticides precisely where and when needed, minimizing environmental impact. When combined with digital farming technologies, they enable precise application methods that reduce wastage and enhance pest management (Atanda et al., 2025).

Nanosensors: Vital tools engineered to detect biological molecules and heavy metal concentrations, helping maintain optimal growth conditions and prevent contamination. They increase sensitivity and selectivity in gas detection, enabling real-time monitoring of environmental health indicators (Shinde et al., 2024).

Nanomediators: Advanced carriers that enable precise delivery of genetic material, allowing plants to regulate gene expression and respond effectively to stresses. They facilitate controlled nutrient release and reduce residual organic pollutants (Rajput et al., 2023; Mgadi et al., 2024).

#### 5.1.4 Seed priming and stress memory enhancement

Recent studies explore NPs’ potential to improve stress memory in seeds through brief exposure to stress stimuli, triggering faster induction of stress-related signaling pathways upon subsequent exposures. This results in enhanced germination and growth under stressful conditions (Khan et al., 2023).

Metal nanoparticles (Ag, Cu, Ti, Au, Zn, Fe) and their oxides, synthesized via eco-friendly methods, significantly increase germination rates and promote plant growth (Riseh and Vazvani, 2024). For example, nano-primed stevia seedlings exhibited 106% increased germination percentage and 128.12% increased germination speed, with seedling dry weights increasing by 283%, 168.9%, and 220% for roots, shoots, and total biomass, respectively (Eevera et al., 2023; Mazhar et al., 2022a).

Multi-walled carbon nanotubes (MWCNTs) at 100 μg/mL enhanced germination and seedling growth in barley, soybean, and maize, while silicon nanoparticles (SiNPs) at 20 ppm improved growth and photosynthetic efficiency in common beans (Naidu et al., 2023).

#### 5.1.5 Sustainable agriculture and environmental benefits

Nanotechnology reduces adverse effects of conventional farming by improving pesticide and fertilizer efficiency, minimizing chemical applications, and consequently decreasing risks of soil erosion, nutrient loss, and pesticide pollution. This promotes biodiversity conservation and enhances agricultural ecosystem sustainability (Nawaz et al., 2023).

The technology offers complementary solutions to conventional breeding limitations by supporting genetic improvement through targeted modifications and nanomaterials that promote plant resistance, leading to crops capable of withstanding climate change and abiotic stresses (Tortella et al., 2023).

#### 5.1.6 Long-term sustainability considerations

The accumulation of NPs in soil ecosystems requires careful assessment of long-term sustainability. While improving plant resilience, they influence microbial diversity, soil enzymatic activities, and soil–plant interactions (MacDonald and Mohan, 2025). Extensive long-term field studies are essential to evaluate NP behavior under different soil types and climatic conditions, ensuring nanotechnology benefits are realized without compromising ecosystem health (Sundararajan et al., 2023).

### 5.2 Traditional vs. nanotechnology approaches in agriculture: a comprehensive comparison

The global agricultural system faces unprecedented challenges, including climate change, soil degradation, water scarcity, and the need to feed a growing population while minimizing environmental impact (FAO (Food and Agriculture Organization), 2021). This demands innovative solutions that can address multiple constraints simultaneously. While traditional agricultural methods have proven reliable over decades, their limitations in terms of efficiency and environmental impact are increasingly apparent. Simultaneously, nanotechnology emerges as a promising alternative, offering precision and enhanced efficacy in crop stress management (Nandeha et al., 2025).

Traditional methods, including the use of conventional fertilizers, pesticides, and selective breeding, have supported agricultural productivity for decades. These methods demonstrate consistent performance across diverse environmental conditions and crop systems, with urea fertilizer application increasing global cereal production by an estimated 48% since 1961 (Stewart et al., 2004).

Furthermore, traditional pesticides and fertilizers operate within well-established regulatory frameworks that encompass comprehensive safety databases spanning decades of use. This regulatory maturity provides farmers and consumers with confidence in application and safety profiles.

Traditional approaches suffer from significant efficiency limitations. Research consistently demonstrates low nutrient use efficiency in conventional systems, with nitrogen use efficiency (NUE) in cereal crops typically ranging from 30 to 50% addition to significant losses through volatilization, leaching, and denitrification (Raun and Johnson, 1999). Similarly, traditional approaches have shown limited improvements in water use efficiency, achieving only (10-25)% improvements under stress conditions.

In contrast, Nanotechnology addresses many limitations of traditional methods through precision in nutrient delivery. Nanoparticles offer size-dependent cellular uptake mechanisms, with studies demonstrating that nanoparticles between 10 and 40 nm in diameter exhibit optimal cellular penetration in plant tissues (Schwab et al., 2016). This precision is exemplified by zinc oxide nanoparticles (ZnO-NPs) at 25 nm, which showed 85% higher zinc uptake efficiency in tomato plants compared to conventional zinc sulfate, with targeted accumulation in chloroplasts where zinc is most needed for photosynthetic enzymes (Raliya et al., 2015).

Unlike traditional single-purpose applications, single nanoparticle formulations can address multiple stress factors simultaneously through engineered multi-functionality (Mittal et al., 2020). Cerium oxide nanoparticles (CeO_2_-NPs) demonstrate this dual functionality as both antioxidants and photosynthetic enhancers. In drought-stressed soybean plants, CeO_2_-NPs (3nm) increased antioxidant enzyme activity by 45% while maintaining photosynthetic efficiency at 80% of well-watered controls (Wu et al., 2017).

Nanotechnology enables a significant reduction in chemical inputs while maintaining or improving efficacy (Fraceto et al., 2016). Nano-encapsulated herbicides require 60-80% lower active ingredient concentrations compared to conventional formulations while achieving equivalent weed control, reducing environmental contamination risk (Kumar et al., 2019). Additionally, the integration of nanosensors enables continuous monitoring and responsive treatment systems (Giraldo et al., 2019), with carbon nanotube-based sensors embedded in plant leaves detecting nitric oxide levels in real-time and triggering automated delivery of protective nanoparticles when stress thresholds are exceeded (Wang X. et al., 2023).

Direct comparisons reveal substantial advantages for nanotechnology approaches. Studies comparing nano-fertilizers to conventional alternatives showed average nutrient use efficiency improvements of (35-65) % across major nutrients (Liu and Lal, 2015). Water Use Efficiency improvement is even more dramatic, with nanoparticle applications achieving (25-60) % improvement compared to the traditional method. For instance, silicon nanoparticles (Si-NPs) applied to wheat under drought stress improved grain yield by 45% compared to 18% improvement with conventional silicon fertilizer (Frazier et al., 2014).

In addition, nanotechnology is demonstrating superior performance in enhancing plant stress tolerance. Iron oxide nanoparticles (Fe_2_O_3_NPs) enhanced salt tolerance in barley, maintaining 75% of normal yield under less than 150 mM NaCl stress compared to 45% with conventional treatments (Mukhtiar et al., 2024). However, these benefits come with complexity, as the same treatments can have variable effects across different plant systems.

High production costs are a significant barrier to the adoption of nanotechnology. Nano-fertilizer production costs range from 2,000 to 8,000 $ per ton compared to 400 to 800 $ for conventional fertilizers (Subramanian et al., 2015). This economic gap is more pronounced for specialized applications, with silver nanoparticles for antimicrobial applications costing approximately $50-200 per gram, making large-scale agricultural applications economically prohibitive (Sharma et al., 2018).

The global nano-fertilizer market is projected to reach $4.8 billion by 2025, growing at a 12.3% CAGR, driven by precision agriculture adoption (Grand View Research, 2020). This growth trajectory suggests increasing commercial viability as production scales increase and costs decrease.

Rather than viewing nanotechnology as a complete replacement for traditional methods, an integrated approach offers optimal benefits. This strategy involves using nanoparticles for high-value crops or specific stress conditions where cost-benefit ratios are favorable, combining nano-enhanced precision delivery with conventional breeding and management practices, and implementing phase-wise adoption with continuous monitoring and adaptive management.

### 5.3 Nanotechnology-enhanced sensing and modeling for precision plant stress management

Several non-AI predictive technologies are crucial for detecting and managing plant abiotic stress. Biosensors and nanobiosensors are primary examples. Nanomaterials possess unique and tunable properties, such as optical characteristics, electrical conductivity, and shock resistance. These properties enable the creation of flexible and highly sensitive detection mechanisms capable of measuring a broad spectrum of physiological and environmental parameters relevant to plant stress (Mukherjee et al., 2024). These sophisticated analytical systems integrate a biological sensing element with a transducer, enabling the detection of specific biomarkers that indicate stress in plants. Nanotechnology significantly enhances the effectiveness of these biosensors by boosting their sensitivity and specificity, thereby facilitating the early and precise identification of both abiotic and biotic stresses (Rana et al., 2024).

Biosensors and nanobiosensors play a crucial role in the timely identification of stress factors. Their early detection capabilities are due to proactive interventions, which are critical in mitigating potential crop losses before they become severe. The incorporation of nanomaterials in biosensor development allows for a significant increase in sensitivity and overall efficacy (Tang et al., 2024). This enables the detection of even trace amounts of stress-related biomarkers, providing a more accurate and nuanced understanding of plant stress (Sheikh et al., 2024). Nanobiosensors are versatile and capable of detecting a wide array of biological derivatives. This includes the identification of bacteria, viruses, and various chemical toxins that can impact agricultural produce, thereby contributing to food safety and quality control. These tools are often integrated with continuous monitoring platforms, allowing for real-time data collection on plant physiological responses to environmental changes (Chaturvedi et al., 2025).

Some models used to study the behavior of NPs and their effects on plants foe example 1- Computational Modeling: Simulating NP transport in plant tissues (e.g., using finite element analysis). 2- Physiologically Based Pharmacokinetic (PBPK) Models: Predicting NP fate within plants based on plant physiology (Huang et al., 2024). 3- Quantitative Structure-Activity Relationships (QSAR): Relating NP properties (size, charge, coating) to biological effects (uptake, toxicity, and efficacy). 4- Kinetic Modelling: Predicting NP dissolution rates and ion release over time in the rhizosphere. 5- Stochastic Models: Assessing variability in NP behavior and plant responses under field conditions (Yu et al., 2023; Thiruvengadam et al., 2025; Sarabandi et al., 2025). These technologies empower farmers and researchers to implement targeted management strategies, advancing toward a more responsive and efficient agricultural system without relying on complex AI models for prediction. Recent studies indicate that the use of single-walled carbon nanotube (SWNT) nano-sensors for real-time detection of SA and H_2_O_2_, elucidating stress-specific biochemical waveforms, and enabling early interventions to promote stress tolerance. Other nano-sensors can measure nutrient levels, pathogen attacks, or pollutant stress, further supporting crop resilience under abiotic (drought, salinity, heat) stress conditions. While promising, challenges remain regarding nanosensor stability, long-term effects on plants, potential environmental impacts, and cost-effectiveness for large-scale field use.

## 6 The challenges of integrating nanotechnology in agriculture

### 6.1 Environmental concerns and long-term safety assessment of nanoparticles in agriculture

The rapid advancement of agricultural nanotechnology has generated considerable enthusiasm for its potential to address global food security challenges while improving environmental sustainability (Thakur and Yadav, 2025). However, the unique physicochemical properties that make nanoparticles effective in agricultural applications also raise concerns about their environmental fate, ecological impacts, and long-term safety implications. The limited understanding of nanoparticle behavior in complex environmental systems necessitates a precautionary approach that balances innovation with environmental protection (Gupta, 2021).

Current knowledge gaps regarding the long-term environmental consequences of nanoparticle applications in agriculture highlight the urgent need for comprehensive risk assessment frameworks, enhanced monitoring systems, and adaptive regulatory approaches. This assessment examines the current state of knowledge regarding environmental risks while identifying critical research priorities and policy needs for sustainable implementation of agricultural nanotechnology (**Table 5**) (Thakur and Yadav, 2025).

**Table 5:** Nanotoxicity in plants: mechanisms and its effects.

Nanoparticle type	Toxicity mechanisms	Physiological/morphological effects	Reference
ZnO NPs	ROS overproduction, disruption of antioxidant systems, DNA damage; accumulation via apoplastic/symplastic paths	Inhibited germination, stunted root/shoot, chlorosis, lipid peroxidation, genotoxicity	(Kang et al., 2024)
TiO_2_ and other metallic NPs	Light-induced ROS, chloroplast dysfunction, membrane damage	Reduced photosynthesis, cellular structural damage	(Zhang et al., 2025)
Silicon NPs	Block uptake of trace metals; modulation of antioxidant defenses and gene expression	Enhanced stress tolerance, reduced metal toxicity	(Mukarram et al., 2024)
Nanoplastics	Foliar penetration, disruption of phyllosphere microbes; elevated oxidative enzymes, altered metabolism	Inhibited growth, altered gene expression, microbial community shifts	(Shi et al., 2024)
Combined NPs and Heavy Metals	Synergistic or antagonistic interactions affecting ROS, ion homeostasis	Reduced heavy metal uptake, improved enzyme activity, biomass recovery	(Soni et al., 2024)
Carbon-based Quantum Effects	Membrane interaction, DNA methylation changes, ROS induction	Stomatal closure, germination delay, transcriptomic/proteomic changes	(Gowtham et al., 2024)

### 6.2 Soil ecotoxicity and ecosystem impact assessment

#### 6.2.1 Microbial community disruption and functional alterations

Soil microbial communities represent the foundation of terrestrial ecosystem functioning, mediating critical processes, including nutrient cycling, organic matter decomposition, and plant-microbe interactions. Nanoparticles can significantly alter soil microbial diversity and functionality through direct toxic effects, modification of soil chemistry, and interference with microbial communication systems (Kumar et al., 2025).

Silver nanoparticles (AgNPs) demonstrate broad-spectrum antimicrobial activity that, while beneficial for pathogen control, can indiscriminately affect beneficial soil microorganisms, including nitrogen-fixing bacteria such as Rhizobium species, mycorrhizal fungi (Glomus spp.), and decomposer organisms essential for nutrient mineralization (Sillen et al., 2015). Laboratory studies indicate that AgNPs at concentrations of 1-10 mg/kg soil reduce bacterial diversity by 15-40% within 30 days of application, with effects persisting for several months after initial exposure (Das and Ingti, 2025).

#### 6.2.2 Enzymatic activity suppression

Key soil enzymes, including dehydrogenase (an indicator of overall microbial activity), phosphatase (phosphorus mineralization), and β-glucosidase (carbon cycling), show reduced activity ranging from 20-60% inhibition following exposure to metal oxide nanoparticles at environmentally relevant concentrations. Copper oxide nanoparticles (CuO NPs) demonstrate particular concern for nitrogen cycling, inhibiting nitrification processes by 25-50% and affecting nitrogen availability for plant uptake (Bouhadi et al., 2025).

#### 6.2.3 Mycorrhizal association disruption

Titanium dioxide nanoparticles (TiO_2_ NPs) interfere with arbuscular mycorrhizal fungi (AMF) colonization, reducing plant nutrient uptake efficiency by 10-30% and compromising the symbiotic relationships essential for sustainable agriculture. These effects are particularly concerning given the critical role of mycorrhizal associations in phosphorus acquisition and plant stress tolerance (Das and Ingti, 2025).

#### 6.2.4 Soil chemical properties and geochemical alterations

Nanoparticles can fundamentally alter soil chemistry through various mechanisms, including ion release, surface reactions, and modification of soil colloid properties. Zinc oxide nanoparticles (ZnO NPs) dissolve preferentially in acidic soils, releasing Zn^2+^ ions that increase soil pH and affect the bioavailability of other nutrients, including phosphorus, iron, and manganese (Li et al., 2025).

Iron-based nanoparticles can reduce soil pH through oxidation reactions and electron transfer processes, potentially mobilizing heavy metals and altering phosphorus availability through changes in adsorption-desorption equilibria. These geochemical alterations may have long-lasting effects on soil fertility and plant nutrient availability, requiring careful monitoring and management (Das and Ingti, 2025).

#### 6.2.5 Organic matter interactions

Nanoparticles demonstrate strong binding affinity for soil organic matter (SOM) through electrostatic interactions, hydrophobic associations, and coordination bonding. This binding can either stabilize organic carbon by protecting it from microbial decomposition (positive effect) or interfere with natural decomposition processes by blocking enzyme access or altering microbial community structure (negative effect), with significant long-term implications for soil carbon sequestration and fertility maintenance (Khort et al., 2021).

### 6.3 Environmental persistence and bioaccumulation assessment

#### 6.3.1 Nanoparticle fate and transformation in soil systems

Understanding the environmental fate of nanoparticles requires a comprehensive evaluation of their persistence, transformation, and mobility in soil systems under varying environmental conditions (Wei et al., 2024). Metal nanoparticles, particularly silver and copper-based formulations, demonstrate high persistence in soil with half-lives ranging from 6 months to several years depending on soil pH, organic matter content, moisture levels, and microbial activity (Das and Ingti, 2025).

Metal oxide nanoparticles, including TiO_2_ and ZnO, demonstrate extreme environmental persistence with minimal degradation over 2-5 year monitoring periods, raising concerns about long-term accumulation and potential ecological effects. Carbon-based nanoparticles, including fullerenes and carbon nanotubes, show variable persistence ranging from months to years, with degradation rates influenced by soil organic content, microbial diversity, and environmental conditions (Zaman et al., 2025).

Nanoparticles undergo various transformation processes in soil environments that affect their bioavailability, toxicity, and environmental fate. Dissolution processes result in the release of ionic forms, particularly relevant for Ag, Cu, and Zn nanoparticles, which may exhibit different toxicological profiles compared to their nano-form counterparts (Das and Ingti, 2025).

Aggregation and agglomeration processes lead to the formation of larger particle clusters that alter surface area-to-volume ratios, reducing bioavailability while potentially affecting soil physical properties. Surface coating with natural organic matter modifies nanoparticle surface properties, potentially reducing toxicity while affecting transport and bioavailability (Khort et al., 2021).

#### 6.3.2 Bioaccumulation potential and trophic transfer

Plant uptake and translocation studies demonstrate variable patterns across plant species and nanoparticle types, with implications for food chain contamination and human exposure. Root accumulation typically represents the highest concentration point, with 50-90% of absorbed nanoparticles retained in root tissues rather than translocated to aerial plant parts (Isibor et al., 2024).

Shoot translocation remains limited for most nanoparticle types, with only 5-30% of absorbed nanoparticles detected in above-ground tissues, though this varies significantly with nanoparticle size, surface coating, and plant species. Grain and fruit accumulation generally shows low but detectable levels (0.1-5% of absorbed nanoparticles) in edible tissues, raising questions about long-term dietary exposure implications (Alizadeh et al., 2025).

Soil invertebrates, including earthworms (*Eisenia fetida*) and springtails (*Folsomia candida*), demonstrate significant nanoparticle accumulation with bioconcentration factors ranging from 1.2 to 3.5, indicating potential for trophic transfer to higher levels. Limited studies on secondary consumers suggest potential transfer to birds and small mammals, though transformation and reduced bioavailability may limit bioaccumulation compared to persistent organic pollutants (Waalewijn-Kool et al., 2014).

### 6.4 Phytotoxicity assessment at environmentally relevant concentrations

#### 6.4.1 Field application and physiological disruption

Field-scale concentrations of nanoparticles often differ significantly from laboratory test conditions, requiring careful evaluation of effects at environmentally relevant exposure levels. Even at low concentrations (0.1-10 mg/L in soil solution), nanoparticles can induce oxidative stress through increased reactive oxygen species (ROS) production, leading to cellular damage and reduced plant performance (Das and Ingti, 2025).

Membrane integrity disruption affects nutrient and water uptake efficiency, with consequences for plant growth and stress tolerance under field conditions. Photosynthetic efficiency reductions of 5-25% have been documented at nanoparticle concentrations of 1-50 mg/kg soil, with implications for carbon fixation and overall plant productivity.

Root development inhibition, including reduced elongation and branching, affects nutrient acquisition capacity and may compromise plant establishment and growth under field conditions (**Table 5**) (Djanaguiraman et al., 2024).

#### 6.4.2 Species-specific sensitivity and agricultural implications

Crop sensitivity to nanoparticles varies significantly among species and cultivars, requiring species-specific risk assessment approaches. Leguminous crops, including soybean and pea, demonstrate high sensitivity due to their dependence on rhizobial nitrogen-fixing symbionts that may be disrupted by nanoparticle exposure.

Leafy vegetables, including lettuce and spinach, show rapid nanoparticle uptake through extensive root systems, potentially leading to higher accumulation levels in edible tissues. Cereal crops, including wheat and rice, demonstrate moderate sensitivity with significant species-specific variations in uptake, translocation, and toxic response thresholds (Raza et al., 2025).

#### 6.4.3 Field-scale reality versus laboratory assessments

Laboratory studies often employ nanoparticle concentrations 10-1000 times higher than realistic field applications, potentially overestimating toxicity risks. However, localized concentration hotspots near application sites can reach levels of concern, with soil surface accumulation showing 2-10 times higher concentrations in the top 5 cm compared to deeper soil layers.

Rhizosphere concentrations may be 1.5-5 times higher than bulk soil levels due to root exudate interactions and localized accumulation processes. Seasonal variations in nanoparticle concentrations result from leaching, plant uptake, microbial degradation, and reapplication cycles, requiring long-term monitoring to understand exposure patterns (Yang et al., 2025).

## 7 Regulation and sustainability

### 7.1 Regulatory frameworks and policy gap analysis

#### 7.1.1 Current regulatory inadequacies

Existing pesticide and fertilizer regulations prove inadequate for nanomaterials due to their focus on chemical composition rather than size-dependent properties that determine nanoparticle behavior and effects. Traditional regulations fail to account for unique nano-scale properties, including high surface area-to-volume ratios, quantum effects, and enhanced reactivity that distinguish nanoparticles from their bulk counterparts (Bouhadi et al., 2025).

Standard ecotoxicity testing protocols may not capture nano-specific effects such as particle-cell interactions, intracellular uptake mechanisms, and subcellular localization that influence toxicity profiles. Current regulatory approval processes rely on short-term studies spanning weeks to months rather than the long-term assessments (years to decades) necessary to evaluate environmental persistence and chronic exposure effects (Yang et al., 2025).

#### 7.1.2 International regulatory inconsistencies

The European Union has implemented the most comprehensive regulatory approach through the REACH (Registration, Evaluation, Authorization, and Restriction of Chemicals) regulation, which requires the submission of nano-specific data for nanomaterials. However, implementation remains inconsistent across member states, and enforcement mechanisms require strengthening (Gupta et al., 2023).

The United States Environmental Protection Agency (EPA) continues developing nano-specific guidance documents, but implementation remains inconsistent across different agricultural applications and regulatory pathways. Developing countries face significant challenges in building regulatory capacity for nanotechnology oversight, which can create international trade implications, including the lack of harmonized standards creating trade barriers, regulatory shopping where manufacturers seek the least restrictive jurisdictions, and insufficient coordination on global risk assessment approaches (Yang et al., 2025).

#### 7.1.3 Critical regulatory development needs

Priority regulatory developments include nano-specific risk assessment protocols that account for size, surface area, reactivity, and environmental fate characteristics unique to nanomaterials. Standardized characterization requirements should mandate comprehensive physical-chemical characterization, including particle size distribution, surface area, surface charge, and coating composition for all nano-enabled agricultural products (Mittal et al., 2020).

Long-term environmental monitoring requirements should establish post-market surveillance systems for tracking environmental concentrations, ecosystem effects, and bioaccumulation patterns over extended periods. Lifecycle assessment integration should consider environmental impacts across production, use, and disposal phases of nanomaterial lifecycles (Yang et al., 2025).

### 7.2 Risk mitigation strategies and sustainable implementation

#### 7.2.1 Design-based risk reduction approaches

Biodegradable nanoparticle development focuses on creating environmentally degradable formulations that maintain agricultural efficacy while reducing persistence concerns. Targeted delivery systems enable precision application strategies that minimize environmental exposure while maximizing agricultural benefits.

Surface modification techniques can reduce toxicity while maintaining efficacy through coatings that control release rates, reduce bioavailability, or enhance biodegradation. Temporal application strategies involve timing applications to minimize ecological impact during sensitive periods such as pollinator activity, spawning seasons, or critical growth phases (Suresh Kumar et al., 2025).

#### 7.2.2 Environmental monitoring and adaptive management

Environmental monitoring frameworks should include baseline assessment protocols for characterizing pre-application environmental conditions, real-time monitoring systems for continuous assessment of soil and water quality, and biological indicator programs using sensitive species as early warning systems.

Adaptive management protocols should establish clear criteria for modifying application practices based on monitoring results, stakeholder engagement processes for incorporating community concerns and scientific findings, and regular review cycles for updating risk assessments based on new scientific evidence (Meng et al., 2025).

### 7.3 Recommendations for responsible development

#### 7.3.1 Integrated risk-benefit framework

The implementation of the precautionary principle necessitates adopting conservative measures until comprehensive safety data is available. Concurrently, stakeholder engagement ensures the involvement of farmers, environmental groups, consumers, and regulatory agencies in the decision-making process. This can be achieved by transparent communication, which involves openly sharing research findings, risk assessments, and acknowledgments of uncertainty with all parties involved (Zaman et al., 2025).

#### 7.3.2 Research priority identification

Critical research needs include long-term field studies spanning multiple years and diverse environmental conditions, mechanistic toxicity studies elucidating cellular and molecular mechanisms of nanoparticle effects, and ecosystem-level studies examining community and population-level responses to chronic exposure (Meng et al., 2025).

Standardized testing protocol development should establish harmonized methods for nanoparticle characterization, environmental fate assessment, and ecological risk evaluation. International cooperation initiatives should promote shared research programs, harmonized regulatory standards, and technology transfer to developing countries (Das and Ingti, 2025).

### 7.4 Long-term environmental sustainability assessment

#### 7.4.1 Ecosystem-level impact evaluation

Chronic exposure to nanoparticles could induce changes at the ecosystem level. These changes may include a reduction in biodiversity due to the preferential toxicity of nanoparticles to sensitive species, shifts in functional groups that affect decomposer and nutrient cycling communities, and the development of resistance in exposed organisms. Consequently, these alterations have the potential to disrupt essential ecosystem services, such as pollination, biological pest control, and nutrient cycling, which are critical for maintaining sustainable agricultural systems (Vita et al., 2023).

#### 7.4.2 Cumulative and interactive effects assessment

Nanoparticles interact synergistically with other environmental stressors, including climate change, where changes in temperature and moisture affect nanoparticle behavior and toxicity profiles. Chemical pollution interactions may result in additive or synergistic effects when nanoparticles combine with pesticides, heavy metals, or other contaminants.

Habitat fragmentation reduces ecosystem resilience to nanoparticle stress, while agricultural intensification creates multiple stressor scenarios where nanoparticles interact with conventional agricultural inputs (Meng et al., 2025).

## 8 Conclusion and future perspectives

Nanotechnology presents a promising tool for advancing sustainable agriculture. It enhances plant tolerance to abiotic stresses, such as salinity and drought, improves nutrient uptake efficiency, and bolsters natural defense mechanisms. Furthermore, nanobiosensors enable the early detection of environmental stressors, while green synthesis methods offer an environmentally friendly production pathway for nanoparticles. However, a thorough understanding of nanoparticle interactions with plant systems and the environment, particularly those involving complex biological processes, remains essential.

A comparison with traditional approaches reveals significant advantages for nanotechnology in terms of efficiency, precision, and a reduced environmental footprint. Nevertheless, major challenges such as high production costs, safety concerns, and regulatory gaps must be addressed for its successful implementation. Evidence suggests that relying on any single approach is insufficient to meet modern agricultural demands; instead, the future lies in the intelligent integration of nanotechnology with traditional practices to develop productive and sustainable farming systems.

Realizing this potential while minimizing risks requires a responsible, research-driven strategy. This must include broad stakeholder engagement, adaptive management practices, and robust regulatory frameworks to ensure safe and equitable adoption. Environmental concerns and long-term safety issues demand urgent scientific attention and proactive policy development. Implementing comprehensive risk-assessment frameworks, establishing robust environmental monitoring programs, and fostering international cooperation will be critical to ensuring that nanotechnology benefits agriculture without compromising human or ecosystem health.

Looking ahead, research priorities should focus on developing sustainable synthesis methods, designing smart delivery systems, and elucidating the molecular mechanisms of nanoparticle-plant interactions. Integrating nanotechnology with fields such as genomics, proteomics, and metabolomics will provide a holistic understanding of plant responses to stress, which is essential for developing climate-resilient crops. Through this interdisciplinary, precautionary, and integrated approach, nanotechnology can significantly boost crop yields, even on marginal lands, thereby contributing to global food security while protecting environmental health.
