# Correction: Crop management to enhance plant resilience to abiotic stress using nanotechnology: towards more efficient and sustainable agriculture

**DOI:** 10.3389/fpls.2025.1728599

**Published:** 2025-12-04

**Authors:** Othman Al-Dossary, Lina M. Alnaddaf, Jameel M. Al-Khayri

**Affiliations:** 1 Department of Agricultural Biotechnology, College of Agriculture and Food Sciences, King Faisal University, Al-Ahsa, Saudi Arabia; 2 Department of Field Crops, College of Agriculture, Homs University, Homs, Syria

**Keywords:** crop management, signaling pathways, nanotechnology, abiotic stress, sustainable agriculture

In the published article, there was a mistake in the **Acknowledgments** statement. The **Acknowledgments** statement was displayed as:

“The authors extend their appreciation for the support of the Deanship of Scientific Research, Vice Presidency for Graduate Studies and Scientific Research, King Faisal University, Saudi Arabia [Grant No. KFU000000].”

The correct statement is:

“The authors extend their appreciation for the support of the Deanship of Scientific Research, Vice Presidency for Graduate Studies and Scientific Research, King Faisal University, Saudi Arabia [Grant No. KFU253401].’’

The original version of this article has been updated.

